# Factor analysis models via I-divergence optimization

**DOI:** 10.1007/s11336-015-9486-5

**Published:** 2015-11-25

**Authors:** Lorenzo Finesso, Peter Spreij

**Affiliations:** 1 IEIIT - CNR, Via Gradenigo, 6-a, 35129 Padua, Italy; 2 Korteweg-de Vries Institute for Mathematics, Universiteit van Amsterdam, POBox 94248, 1090 GE Amsterdam, The Netherlands

**Keywords:** factor analysis, I-divergence, optimal approximate model, alternating minimization, 62H25, 62B10

## Abstract

Given a positive definite covariance matrix $\widehat{\Sigma }$ of dimension *n*, we approximate it with a covariance of the form $HH^\top +D$, where *H* has a prescribed number $k<n$ of columns and $D>0$ is diagonal. The quality of the approximation is gauged by the I-divergence between the zero mean normal laws with covariances $\widehat{\Sigma }$ and $HH^\top +D$, respectively. To determine a pair (*H*, *D*) that minimizes the I-divergence we construct, by lifting the minimization into a larger space, an iterative alternating minimization algorithm (AML) à la Csiszár–Tusnády. As it turns out, the proper choice of the enlarged space is crucial for optimization. The convergence of the algorithm is studied, with special attention given to the case where *D* is singular. The theoretical properties of the AML are compared to those of the popular EM algorithm for exploratory factor analysis. Inspired by the ECME (a Newton–Raphson variation on EM), we develop a similar variant of AML, called ACML, and in a few numerical experiments, we compare the performances of the four algorithms.

## Introduction

Let *Y* be a given zero mean normal vector of dimension *n* and covariance ${\mathbb {C}}\hbox {ov}(Y)=\widehat{\Sigma }$. A standard Factor Analysis (FA) model for *Y* is a linear model

1.1 $$\begin{aligned} Y = HX + \varepsilon , \end{aligned}$$

where *H* is a deterministic matrix, *X* is a standard normal vector of dimension $k<n$, i.e., with zero mean and ${\mathbb {C}}\hbox {ov}(X)=I_k$ (the *k*-dimensional identity), and $\varepsilon$ is a zero mean normal vector, independent from *X*, with ${\mathbb {C}}\hbox {ov}(\varepsilon )=D$ diagonal. The model (1.1) *explains* the *n* components of *Y* as linear combinations of the $k<n$ components of *X*, perturbed by the independent noise $\varepsilon$. The FA model built-in linear structure and *data reduction* mechanism make it very attractive in applied research.

It is not always possible to describe the given *Y* with a FA model. Indeed, as a consequence of the hypotheses on *X* and $\varepsilon$,

1.2 $$\begin{aligned} \widehat{\Sigma }= HH^\top +D, \end{aligned}$$

a relation which imposes strong structural constraints on the covariance $\widehat{\Sigma }$. Determining whether the given *Y* admits a FA model (1.1) requires the solution of an algebraic problem: given $\widehat{\Sigma }$, find, if they exist, pairs (*H*, *D*) such that (1.2) holds. The structural constraints impose that *H* must be a tall matrix, and *D* a diagonal matrix. For a given $\widehat{\Sigma }$, the existence and uniqueness of a pair (*H*, *D*) are not guaranteed. Generically, the Ledermann bound (Anderson & Rubin, 1956; Ledermann, 1937), gives necessary conditions for the existence of a FA model in terms of *k* and *n*.

As it turns out, for the *data reduction* case of this paper, the right tools to deal with the existence and the construction of an FA model are geometric in nature and come from the theory of stochastic realization, see Finesso and Picci (1984) for an early contribution on the subject.

In the present paper we address the problem of constructing an approximate FA model of the given *Y*. Since in general the relation (1.2) does not hold for any (*H*, *D*), one has to find ways to gauge the closeness of $\widehat{\Sigma }$ to the FA model covariance $HH^\top +D$. One possibility is to use a form of least squares as a loss criterion. Here we adopt the I-divergence ${\mathcal {I}}(\widehat{\Sigma } || HH^\top +D)$, also known as Kullback-Leibler distance, between the corresponding (multivariate) normal laws. Throughout the paper $\widehat{\Sigma }$ is given and is assumed to be non-singular.

In statistical inference it is well known, and reviewed in Section 2, that the I-divergence is, up to constants independent of *H* and *D*, the parameters yielding the covariance $HH^\top + D$, the opposite of the normal log likelihood. One has the identity

1.3 $$\begin{aligned} -{\mathcal {I}}(\widehat{\Sigma } || HH^\top +D)=\frac{n}{2} -\frac{1}{2}\log \frac{|HH^\top +D|}{\widehat{\Sigma }}-\frac{1}{2}\hbox {tr}\Big ((HH^\top + D)^{-1}\widehat{\Sigma }\Big ), \end{aligned}$$

where $\widehat{\Sigma }$ is now the empirical covariance matrix, used as an estimator of the true covariance $HH^\top + D$. In the empirical context non-singular $\widehat{\Sigma }$ is usually the case if the number of variables is smaller than the number of observations. A completely different situation, singular $\widehat{\Sigma }$, arises when the number of variables is larger than the number of observations, see e.g., Bai and Li (2012), Trendafilov and Unkel (2011) for recent results.

The choice of the best (*H*, *D*) pair can then be posed as a maximum- likelihood problem, as first proposed by Lawley (1940). Lacking a closed form solution, the maximization problem (1.3) has to be attacked numerically, and several optimization algorithms have been either adapted or custom-tailored for it. Among the former, the EM method, introduced in the context of FA estimation by Rubin and Thayer (1982), and still mutating and evolving (Adachi, 2013; Zhao, Yu, & Jiang, 2008; Zhao & Shi, 2014), takes full advantage of the structure of the likelihood in order to guarantee descent at each iteration, although at the expense of a less than ideal convergence rate, which can be slow and sensitive to the initial conditions.

It has long been known, see Csiszár and Tusnády (1984), that any EM algorithm can be reformulated embedding the problem in a properly chosen larger parameter space and then performing alternating partial minimizations of the I-divergence over properly defined subspaces. This setup has previously been followed for various problems, e.g., mixture decomposition (Csiszár & Tusnády, 1984), non-negative matrix factorization (Finesso & Spreij, 2006), and approximation of non-negative impulse response functions (Finesso & Spreij, 2015). The advantage afforded by the embedding procedure is that both partial minimizations have closed form solutions; moreover a necessary and sufficient condition of optimality of a geometric flavor, a Pythagoras rule, see Csiszár (1975), is available to check optimality for both partial minimizations. As it turns out, and we prove this assertion in Section 5, the EM method proposed in Rubin and Thayer (1982) corresponds to a suboptimal embedding, as one of the Pythagoras rules fails. The main result of this paper is an iterative algorithm, called AML, for the construction of an (*H*, *D*) pair minimizing the I-divergence from $\widehat{\Sigma }$ using an optimal embedding, for which both Pythagoras rules hold. We also study the behavior of the algorithm in the singular case, i.e., *D* not of full rank, which is well known to be problematic for FA modeling (Jöreskog, 1967). These theoretical considerations make up the bulk of the paper. We emphasize that the present paper is not on numerical subtleties and (often very clever) improvements as established in the literature to accelerate the convergence of EM type algorithms. Rather, the central feature is the systematic methodology to derive an algorithm by a constructive procedure. Nevertheless, we make a brief foray into the numerical aspects, developing a version of AML, which we call ACML, in the spirit of ECME [a Newton–Raphson variation on EM, Liu and Rubin (1994)].

The remainder of the paper is organized as follows. In Section 2 the approximation problem is posed and discussed, as well as its estimation problem counterpart. Section 3 recasts the problem as a double minimization in a larger space, making it amenable to a solution in terms of alternating minimization. In Section 4, we present the alternating minimization algorithm, provide alternative versions of it, and study its asymptotics. We also point out, in Section 5, the similarities and the differences between our algorithm and the EM algorithm. Section 6 is dedicated to a constrained version of the optimization problem (the singular *D* case) and the pertinent alternating minimization algorithm. The study of the singular case also sheds light on the boundary limit points of the algorithm presented in Section 4. The last Section 7 is devoted to numerical illustrations, where we compare the performance of the AML, EM, ACML, and ECME algorithms. The Appendix contains the proofs of most of the technical results, and also decomposition results on the I-divergence, which are interesting in their own right, beyond application to Factor Analysis.

## Problem Statement

In the present section, we pose the approximation problem and discuss the closely related estimation problem and its statistical counterpart.

### Approximation Problem

Consider independent normal, zero mean, random vectors *X* and $\varepsilon$, of respective dimensions *k* and *n*, where $k<n$, and with ${\mathbb {C}}\hbox {ov}(X)=I_k$ and ${\mathbb {C}}\hbox {ov}(\varepsilon )=D$, a diagonal matrix. For any deterministic conformable matrix *H*, the *n* dimensional vector *Y* given by

2.1 $$\begin{aligned} Y=HX + \varepsilon \end{aligned}$$

is called a standard FA model. The matrices (*H*, *D*) are the parameters that identify the model. As a consequence of the hypotheses,

2.2 $$\begin{aligned} {\mathbb {C}}\hbox {ov}(Y) = HH^\top +D. \end{aligned}$$

Given an *n*-dimensional covariance matrix $\widehat{\Sigma }$, one can pose the problem of approximating it with the covariance of a standard FA model, i.e., of finding (*H*, *D*) such that

2.3 $$\begin{aligned} \widehat{\Sigma } \approx HH^\top +D. \end{aligned}$$

In this paper, we pose and solve the problem of finding an optimal approximation (2.3) when the criterion of closeness is the I-divergence (also known as Kullback-Leibler distance) between normal laws. Recall that [see e.g., Theorem 1.8.2 in Ihara (1993)] if $\nu _1$ and $\nu _2$ are two zero mean normal distributions on ${\mathbb {R}}^m$, whose covariance matrices, $\Sigma _1$ and $\Sigma _2$, respectively, are both non-singular, the I-divergence ${\mathcal {I}}(\nu _1||\nu _2)$ takes the explicit form ($|\cdot |$ denotes determinant)

2.4 $$\begin{aligned} {\mathcal {I}}(\nu _1||\nu _2)=\frac{1}{2}\log \frac{|\Sigma _2|}{|\Sigma _1|}-\frac{m}{2} +\frac{1}{2}\mathrm{tr}(\Sigma _2^{-1}\Sigma _1). \end{aligned}$$

Since, because of zero means, the I-divergence depends only on the covariance matrices, we usually write ${\mathcal {I}}(\Sigma _1||\Sigma _2)$ instead of ${\mathcal {I}}(\nu _1||\nu _2)$. The approximate FA model problem can then be cast as follows.

#### **Problem 2.1**

Given the covariance matrix $\widehat{\Sigma }>0$, of size *n*, and an integer $k<n$, minimize^1^

2.5 $$\begin{aligned} {\mathcal {I}}(\widehat{\Sigma } || HH^\top +D) = \frac{1}{2}\log \frac{|HH^\top +D|}{|\widehat{\Sigma }|}-\frac{n}{2} +\frac{1}{2}\mathrm{tr}((HH^\top +D)^{-1}\widehat{\Sigma }), \end{aligned}$$

where the minimization is taken over all diagonal matrices $D\ge 0$, and $H \in {\mathbb {R}}^{n\times k}$.

The first result is that in Problem 2.1, a minimum always exists.

#### **Proposition 2.2**

There exist matrices $H^*\in {\mathbb {R}}^{n\times k}$, and non-negative diagonal $D^*\in {\mathbb {R}}^{n\times n}$ that minimize the I-divergence in Problem 2.1.

#### *Proof*

The proof can be found in Finesso and Spreij (2007). $\square$

Finesso and Spreij (2006) studied an approximate non-negative matrix factorization (NMF) problem where the objective function was also of I-divergence type. In that case, using a relaxation technique, the original minimization was lifted to a double minimization in a higher dimensional space, leading naturally to an alternating minimization algorithm. The core of the present paper consists in following the same approach, in the completely different context of covariance matrices, and to solve Problem 2.1 with an alternating minimization algorithm.

As a side remark note that ${\mathcal {I}}(\Sigma _1||\Sigma _2)$, computed as in (2.4), can be considered as an I-divergence between two positive definite matrices, without referring to normal distributions. Hence the approximation Problem 2.1 is meaningful even without assuming normality.

### Estimation Problem

In a statistical setup, the approximation Problem 2.1 has an equivalent formulation as an *estimation* problem. Let indeed $Y_1,\ldots ,Y_N$ be a sequence of *i.i.d.* observations, whose distribution is modeled according to (2.1), where the matrices *H* and *D* are the unknown parameters. This is the context of Exploratory Factor Analysis, where no constraints are imposed on the matrix *H*. Let $\widehat{\Sigma }$ denote the sample covariance matrix of the data. If the data have strictly positive covariance, for large enough *N*, the sample covariance will be strictly positive almost surely. The normal log likelihood $\ell (H,D)$ yields

2.6 $$\begin{aligned} \frac{1}{N}\ell (H,D)=-\frac{n}{2}\log (2\pi )-\frac{1}{2}\log |HH^\top +D|-\frac{1}{2}\hbox {tr}\Big ((HH^\top + D)^{-1}\widehat{\Sigma }\Big ). \end{aligned}$$

One immediately sees that $\ell (H,D)$ is, up to constants not depending on *H* and *D*, equal to $-{\mathcal {I}}(\widehat{\Sigma }||HH^\top + D)$. Hence, maximum-likelihood estimation parallels I-divergence minimization in Problem 2.1, only the interpretation is different.

The equations for the maximum-likelihood estimators can be found in e.g., Section 14.3.1 of Anderson (1984). In terms of the unknown parameters *H* and *D*, with *D* assumed to be non-singular, they are

2.7 $$\begin{aligned} H&=(\widehat{\Sigma }-HH^\top )D^{-1}H \end{aligned}$$

2.8 $$\begin{aligned} D&=\Delta (\widehat{\Sigma }-HH^\top ). \end{aligned}$$

where $\Delta (M)$, defined for any square *M*, coincides with *M* on the diagonal and is zero elsewhere. Note that the matrix $HH^\top +D$ obtained by maximum-likelihood estimation is automatically invertible. Then it can be verified that Equation (2.7) is equivalent to

2.9 $$\begin{aligned} H =\widehat{\Sigma }(HH^\top +D)^{-1}H, \end{aligned}$$

which is meaningful also when *D* is not invertible.

It is clear that the system of Equations (2.7), (2.8) does not have an explicit solution. For this reason, several numerical algorithms have been devised, among others a version of the EM algorithm, see Rubin and Thayer (1982). In the present paper we consider an alternative approach, which we will compare with the EM and some of the other algorithms.

## Lifted Version of the Problem

In this section, Problem 2.1 is recast in a higher dimensional space, making it amenable to solution via two partial minimizations which will lead, in Section 4.1, to an alternating minimization algorithm. The original *n* dimensional FA model (2.1) is embedded in a larger $n+k$ dimensional linear model as follows:

3.1 $$\begin{aligned} V=\begin{pmatrix} Y \\ Z\end{pmatrix} = \begin{pmatrix}H &{}I_n\\ Q^\top &{} 0\end{pmatrix}\begin{pmatrix} X \\ \varepsilon \end{pmatrix}\,, \end{aligned}$$

where the deterministic matrix $Q^\top$ is square of size *k*, and for the terms *H*, *X*, and $\varepsilon$ the hypotheses leading to model (2.1) still hold. The vector *V*, as well as its subvector *Z*, is therefore zero mean normal, with

3.2 $$\begin{aligned} {\mathbb {C}}\hbox {ov}(V)=\begin{pmatrix} HH^\top + D &{} HQ \\ (HQ)^\top &{} Q^\top Q &{} \end{pmatrix}. \end{aligned}$$

### *Remark 3.1*

The embedding (3.1) has a simple interpretation as a convenient reparametrization of the following alternative version of the standard FA model (2.1),

3.3 $$\begin{aligned} Y=LZ +\varepsilon , \end{aligned}$$

where *Z* and $\varepsilon$ are zero mean normal vectors of sizes *k* and *n*, respectively, with ${\mathbb {C}}\hbox {ov}(Z)=P>0$ and ${\mathbb {C}}\hbox {ov}(\varepsilon )=I_n$. Letting $P=Q^\top Q$ and $X=Q^{-\top }Z$, where *Q* is any $k\times k$ square root^2^ of *P*, one easily recognizes that (3.1) is a reparametrization of (3.3).

In this paper, all vectors are zero mean and normal, with law completely specified by the covariance matrix. The set of all covariance matrices of size $n+k$ will be denoted as ${\varvec{\Sigma }}$. An element $\Sigma \in {\varvec{\Sigma }}$ can always be decomposed as

3.4 $$\begin{aligned} \Sigma =\begin{pmatrix} \Sigma _{11} &{} \Sigma _{12} \\ \Sigma _{21} &{} \Sigma _{22} \end{pmatrix}, \end{aligned}$$

where $\Sigma _{11}$ and $\Sigma _{22}$ are square, of respective sizes *n* and *k*.

Two subsets of ${\varvec{\Sigma }}$, comprising covariance matrices with special structure, will play a major role in what follows. The subset ${\varvec{\Sigma }}_0 \subset {\varvec{\Sigma }}$ contains all covariances (3.4) with $\Sigma _{11}=\widehat{\Sigma }$, a given matrix, i.e.,

$$\begin{aligned} {\varvec{\Sigma }}_0=\{\Sigma \in {\varvec{\Sigma }}: \Sigma _{11}=\widehat{\Sigma }\}. \end{aligned}$$

The generic element of ${\varvec{\Sigma }}_0$ will often be denoted as $\Sigma _0$. Also of interest is the subset ${\varvec{\Sigma }}_1\subset {\varvec{\Sigma }}$, containing all covariances (3.4) of the special form (3.2), i.e.,

$$\begin{aligned} {\varvec{\Sigma }}_1=\left\{ \, \Sigma \in {\varvec{\Sigma }}: \, \Sigma _{11}=HH^\top + D, \,\, \Sigma _{12}=HQ, \, \Sigma _{22}=Q^\top Q \, \hbox { with } \, \, H, D, Q \hbox { as in (3.2) }\right\} . \end{aligned}$$

The generic elements of ${\varvec{\Sigma }}_1$ will often be denoted $\Sigma _1$, or $\Sigma (H,D,Q)$ when the parameters are relevant.

We are now ready to pose the following double minimization problem.

### **Problem 3.2**

Find

$$\begin{aligned} \min _{\Sigma _0\in {\varvec{\Sigma }}_0,\Sigma _1\in {\varvec{\Sigma }}_1}{\mathcal {I}}(\Sigma _0||\Sigma _1). \end{aligned}$$

Problems 3.2 and 2.1 are related by the following proposition.

### **Proposition 3.3**

Let $\widehat{\Sigma }$ be given. It holds that

$$\begin{aligned} \min _{H,D}\, {\mathcal {I}}\left( \widehat{\Sigma }\,||\,HH^\top + D\right) =\min _{\Sigma _0\in {\varvec{\Sigma }}_0,\Sigma _1\in {\varvec{\Sigma }}_1}{\mathcal {I}}\left( \Sigma _0||\Sigma _1\right) . \end{aligned}$$

### *Proof*

The proof can be found in Finesso and Spreij (2007). $\square$

### Partial Minimization Problems

The first partial minimization, required for the solution of Problem 3.2, is as follows.

#### **Problem 3.4**

Given a strictly positive definite covariance matrix $\Sigma \in {\varvec{\Sigma }}$, find

$$\begin{aligned} \min _{\Sigma _0 \in {\varvec{\Sigma }}_0} \, {\mathcal {I}}(\Sigma _0||\Sigma ). \end{aligned}$$

The unique solution to this problem can be computed analytically and is given below.

#### **Proposition 3.5**

The unique minimizer $\Sigma _0^*$ of Problem 3.4 is given by

$$\begin{aligned} \Sigma _0^*=\begin{pmatrix} \widehat{\Sigma } &{} \,\, \widehat{\Sigma }\Sigma _{11}^{-1}\Sigma _{12}\\ \Sigma _{21}\Sigma _{11}^{-1}\widehat{\Sigma } &{} \,\, \Sigma _{22}-\Sigma _{21}\Sigma _{11}^{-1} (\Sigma _{11}-\widehat{\Sigma })\Sigma _{11}^{-1}\Sigma _{12} \end{pmatrix} >0. \end{aligned}$$

Moreover,

3.5 $$\begin{aligned} {\mathcal {I}}(\Sigma _0^*||\Sigma )={\mathcal {I}}(\widehat{\Sigma }||\Sigma _{11}), \end{aligned}$$

and the Pythagorean rule

$$\begin{aligned} {\mathcal {I}}(\Sigma _0||\Sigma )={\mathcal {I}}(\Sigma _0||\Sigma _0^*)+{\mathcal {I}}(\Sigma _0^*||\Sigma ) \end{aligned}$$

holds for any strictly positive $\Sigma _0\in {\varvec{\Sigma }}_0$.

#### *Proof*

See Appendix 2. $$\square $$

Next we turn to the second partial minimization

#### **Problem 3.6**

Given a strictly positive definite covariance matrix $\Sigma \in {\varvec{\Sigma }}$, find

$$\begin{aligned} \min _{\Sigma _1 \in {\varvec{\Sigma }}_1} \, {\mathcal {I}}(\Sigma ||\Sigma _1). \end{aligned}$$

The proposition below gives the explicit solution to this problem.

#### **Proposition 3.7**

A minimizer $\Sigma _1^*=\Sigma (H^*,D^*,Q^*)$ of Problem 3.6 is given by

$$\begin{aligned} Q^*&=\Sigma _{22}^{1/2}\\ H^*&= \Sigma _{12}\Sigma _{22}^{-1/2} \\ D^*&= \Delta (\widetilde{\Sigma }_{11}), \end{aligned}$$

where

$$\begin{aligned} \widetilde{\Sigma }_{11}=\Sigma _{11}-\Sigma _{12}\Sigma _{22}^{-1}\Sigma _{21}. \end{aligned}$$

The corresponding minimizing matrix is

3.6 $$\begin{aligned} \Sigma _1^*=\Sigma (H^*,D^*,Q^*)= \begin{pmatrix} \Sigma _{12}\Sigma _{22}^{-1}\Sigma _{21}+\Delta (\widetilde{\Sigma }_{11}) &{} \Sigma _{12} \\ \Sigma _{21} &{} \Sigma _{22} \end{pmatrix}. \end{aligned}$$

Moreover, ${\mathcal {I}}(\Sigma ||\Sigma _1^*)= {\mathcal {I}}(\widetilde{\Sigma }_{11}||\Delta (\widetilde{\Sigma }_{11}))$ and the Pythagorean rule

3.7 $$\begin{aligned} {\mathcal {I}}(\Sigma ||\Sigma _1)={\mathcal {I}}(\Sigma ||\Sigma _1^*)+{\mathcal {I}}(\Sigma _1^*||\Sigma _1) \end{aligned}$$

holds for any $\Sigma _1=\Sigma (H,D,Q)\in {\varvec{\Sigma }}_1$.

#### *Proof*

See Appendix 2. $$\square $$

Note that Problem 3.6 cannot have a unique solution in terms of the matrices *H* and *Q*. Indeed, if *U* is a unitary $k\times k$ matrix and $H'=HU$, $Q'=U^\top Q$, then $H'H'^\top =HH^\top$, $Q'^\top Q'=Q^\top Q$, and $H'Q'=HQ$. Nevertheless, the optimal matrices $HH^\top$, *HQ*, and $Q^\top Q$ are unique, as it can be easily checked using the expressions in Proposition 3.7.

#### *Remark 3.8*

Note that, since $\Sigma$ is supposed to be strictly positive, $\widetilde{\Sigma }_{11}:=\Sigma _{11}-\Sigma _{12}\Sigma _{22}^{-1}\Sigma _{21}$ is strictly positive too. It follows that $D^*=\Delta (\widetilde{\Sigma }_{11})$ is strictly positive.

We close this section by considering a constrained version of the second partial minimization Problem 3.6 to which we will return in Section 5, when we discuss the connection with the EM algorithm. The constraint that we impose is $Q=Q_0$ fixed, whereas *H* and *D* remain free. The set over which the optimization will be carried out is ${\varvec{\Sigma }}_{10}\subset {\varvec{\Sigma }}_1$ defined as

$$\begin{aligned} {\varvec{\Sigma }}_{10}=\left\{ \, \Sigma \in {\varvec{\Sigma }}\, : \, \Sigma _{11}=HH^\top + D, \,\, \Sigma _{12}=HQ_0, \, \Sigma _{22}=Q_0^\top Q_0 \, \hbox { with } \, \, H, D \hbox { as in } \,(3.2)\, \right\} . \end{aligned}$$

We pose the following constrained optimization problem.

#### **Problem 3.9**

Given a strictly positive covariance $\Sigma \in {\varvec{\Sigma }}$, find

$$\begin{aligned} \min _{\Sigma _{10} \in {\varvec{\Sigma }}_{10}} \, {\mathcal {I}}(\Sigma ||\Sigma _{10}). \end{aligned}$$

The solution is given in the next proposition.

#### **Proposition 3.10**

A solution $\Sigma _{10}^*$ of Problem 3.9 is obtained for $H^*=\Sigma _{12}\Sigma _{22}^{-1}Q_0^\top$, and $D^*$ as in Proposition 3.7, resulting in

3.8 $$\begin{aligned} \Sigma _{10}^*=\begin{pmatrix} \Sigma _{12}\Sigma _{22}^{-1}Q_0^\top Q_0\Sigma _{22}^{-1}\Sigma _{21} + \Delta (\widetilde{\Sigma }_{11}) &{} \Sigma _{12}\Sigma _{22}^{-1}Q_0^\top Q_0 \\ Q_0^\top Q_0\Sigma _{22}^{-1}\Sigma _{21} &{} Q_0^\top Q_0 \end{pmatrix}. \end{aligned}$$

#### *Proof*

See Appendix 2. $$\square $$

For the constrained problem, the Pythagorean rule does not hold. Intuitively, since ${\varvec{\Sigma }}_{10}\subset {\varvec{\Sigma }}_1$ the optimal value of the constrained Problem 3.9 is in general higher than the optimal value of the free Problem 3.6. To compute the extra cost incurred notice that $\Sigma _{10}^* \in {\varvec{\Sigma }}_1$, therefore the Pythagorean rule (3.7) gives

3.9 $$\begin{aligned} {\mathcal {I}}(\Sigma ||\Sigma _{10}^*)= {\mathcal {I}}(\Sigma ||\Sigma _1^*)+{\mathcal {I}}(\Sigma _1^*||\Sigma _{10}^*), \end{aligned}$$

hence ${\mathcal {I}}(\Sigma ||\Sigma _{10}^*)\ge {\mathcal {I}}(\Sigma ||\Sigma _1^*)$, where $\Sigma _1^*$ is as in Proposition 3.7. The quantity ${\mathcal {I}}(\Sigma _1^*||\Sigma _{10}^*)$ represents the extra cost. An elementary computation gives

3.10 $$\begin{aligned} {\mathcal {I}}(\Sigma _1^*||\Sigma _{10}^*)={\mathcal {I}}(\Sigma _{22}||Q_0^\top Q_0), \end{aligned}$$

i.e., the optimizing matrices $\Sigma _1^*$ and $\Sigma _{10}^*$, see (3.6), (3.8), coincide iff $Q_0^\top Q_0=\Sigma _{22}$.

Summarizing the comments on the constrained problem: (i) the optimal value at the minimum is higher since ${\varvec{\Sigma }}_{10}\subset {\varvec{\Sigma }}_1$, (ii) the extra cost is explicitly given by (3.10), as ${\mathcal {I}}(\Sigma _{22}||Q_0^\top Q_0)$, and (iii) there is no analog to the Pythagorean rule (3.7). The conclusion is that the solution $\Sigma _{10}^*$ of the constrained Problem 3.9 is suboptimal for the free Problem 3.6. The consequences of the suboptimality will be further discussed in Section 5.

## Alternating Minimization Algorithm

In this section, the core of the paper, the two partial minimizations of Section 3 are combined into an alternating minimization algorithm for the solution of Problem 2.1. A number of equivalent formulations of the updating equations will be presented and their properties are discussed.

### The Algorithm

We suppose that the given covariance matrix $\widehat{\Sigma }$ is strictly positive definite. To set up the iterative minimization algorithm, assign initial values $H_0, D_0, Q_0$ to the parameters, with $D_0$ diagonal, $Q_0$ invertible and $H_0H_0^\top +D_0$ invertible. The updating rules are constructed as follows. Let $H_t, D_t, Q_t$ be the parameters at the *t*-th iteration, and $\Sigma _{1,t} = \Sigma (H_t,D_t,Q_t)$ the corresponding covariance, defined as in (3.2). Now solve the two partial minimizations as illustrated below.where $\Sigma _{0,t}$ denotes the solution of the first minimization with input $\Sigma _{1,t}$. To express in a compact form the resulting update equations, define

4.1 $$\begin{aligned} R_t=I- H_t^\top (H_tH_t^\top + D_t)^{-1}H_t + H_t^\top (H_tH_t^\top + D_t)^{-1} \widehat{\Sigma } (H_tH_t^\top + D_t)^{-1}H_t. \end{aligned}$$

Note that, by Remark 3.8, $H_tH^\top _t+D_t$ is actually invertible for all *t*, since both $H_0H^\top _0+D_0$ and $Q_0$ have been chosen to be invertible. It is easy to show that also $I- H_t^\top (H_tH_t^\top + D_t)^{-1}H_t$, and consequently $R_t$, are strictly positive and therefore invertible. The update equations resulting from the cascade of the two minimizations are

4.2 $$\begin{aligned} Q_{t+1}&= \Big (Q_t^\top R_t Q_t\Big )^{1/2}, \end{aligned}$$

4.3 $$\begin{aligned} H_{t+1}&= \widehat{\Sigma }(H_tH_t^\top + D_t)^{-1}H_tQ_tQ_{t+1}^{-1}, \end{aligned}$$

4.4 $$\begin{aligned} D_{t+1}&= \Delta (\widehat{\Sigma }-H_{t+1}H_{t+1}^\top ). \end{aligned}$$

Properly choosing the square root in Equation (4.2) will make $Q_t$ disappear from the update equations. This is an attractive feature since, at the *t*-th step of the algorithm, only $H_t$ and $D_t$ are needed to construct the approximation $H_t H_t^\top + D_t$. The choice that accomplishes this is $(Q_t^\top R_t Q_t)^{1/2} = R_t^{1/2} Q_t$, where $R_t^{1/2}$ is a symmetric root of $R_t$, resulting in $Q_{t+1} = R_t^{1/2} Q_t$. Upon substituting $Q_{t+1}$ in Equation (4.3), one gets the AML algorithm.

#### **Algorithm 4.1**

(AML) Given $H_t$, $D_t$ from the *t*-th step, and $R_t$ as in (4.1), the update equations for a I-divergence minimizing algorithm are

4.5 $$\begin{aligned} H_{t+1}&= \widehat{\Sigma }(H_tH_t^\top + D_t)^{-1}H_tR_t^{-1/2} \end{aligned}$$

4.6 $$\begin{aligned} D_{t+1}&= \Delta (\widehat{\Sigma }-H_{t+1}H_{t+1}^\top ). \end{aligned}$$

Since $R_t$ only depends on $H_t$ and $D_t$, see (4.1), the parameter $Q_t$ has been effectively removed from the update equations, although its presence was essential for the derivation.

#### *Remark 4.2*

Algorithm 4.1 has one immediate attractive feature: it preserves at each step the diagonal structure of $\widehat{\Sigma }$. Indeed, if we let $\Sigma _t=H_tH_t^\top +D_t$, then it follows from Equation (4.6) that $\Delta (\Sigma _t)=\Delta (\widehat{\Sigma })$.

Algorithm 4.1 potentially has two drawbacks making its implementation computationally less attractive. To update $H_t$ via Equation (4.5) one has to compute, at each step, the square root of the $k \times k$ matrix $R_t$ and the inverse of the $n \times n$ matrix $H_tH_t^\top + D_t$. The latter problem may in principle be addressed via the matrix inversion lemma, but this requires an invertible $D_t$ which could be problematic in practical situations when one encounters nearly singular $D_t$. An alternative approach to Algorithm 4.1, to avoid the square roots at each iteration, is to update ${\mathcal {H}}_t:=H_tH_t^\top$ and $D_t$ as before.

#### **Proposition 4.3**

Let $H_t$ be as in Algorithm 4.1. Pick ${\mathcal {H}}_0=H_0H_0^\top$, and $D_0$ such that ${\mathcal {H}}_0+D_0$ is invertible. The update equation for ${\mathcal {H}}_{t}$ becomes

4.7 $$\begin{aligned} {\mathcal {H}}_{t+1} = \widehat{\Sigma }({\mathcal {H}}_{t}+D_{t})^{-1}{\mathcal {H}}_{t}\big (D_{t} +\widehat{\Sigma }({\mathcal {H}}_t+D_t)^{-1}{\mathcal {H}}_t\big )^{-1}\widehat{\Sigma }. \end{aligned}$$

#### *Proof*

See Appendix 2. $$\square $$

One can run the update Equation (4.7), for any number *T* of steps, and then switch back to $H_T$, taking any $n\times k$ factor of ${\mathcal {H}}_T$ i.e., solve ${\mathcal {H}}_T = H_T H_T^\top$. Since Equation (4.7) transforms ${\mathcal {H}}_t$ into ${\mathcal {H}}_{t+1}$ preserving the rank, the latter factorization is always possible.

### Asymptotic Properties

In Proposition 4.4 below, we collect the asymptotic properties of Algorithm 4.1, also quantifying the I-divergence decrease at each step.

#### **Proposition 4.4**

For Algorithm 4.1, the followings hold.

- $$H_tH_t^\top \le \widehat{\Sigma }$$ for all $$t\ge 1$$.
- If $D_0>0$ and $\Delta (\widehat{\Sigma } - D_0)>0$ then $D_t>0$ for all $t\ge 1$.
- The matrices $R_t$ are invertible for all $t\ge 1$.
- If $$H_t H_t^\top + D_t = \widehat{\Sigma }$$  then $$H_{t+1}=H_t, \,D_{t+1}=D_t$$.
- Decrease of the objective function:
  $$\begin{aligned} {\mathcal {I}}(\widehat{\Sigma }||\widehat{\Sigma }_t) - {\mathcal {I}}(\widehat{\Sigma }||\widehat{\Sigma }_{t+1})= {\mathcal {I}}(\Sigma _{1,t+1}||\Sigma _{1,t})+{\mathcal {I}}(\Sigma _{0,t}|| \Sigma _{0,t+1}), \end{aligned}$$
  where $\widehat{\Sigma }_t = H_tH_t^\top + D_t$ is the *t*-th approximation of $\widehat{\Sigma }$, and $\Sigma _{0,t}, \Sigma _{1,t}$ were defined in Section 4.1.
- The interior limit points (*H*, *D*) of the algorithm satisfy
  4.8 $$\begin{aligned} H = (\widehat{\Sigma }-HH^\top )D^{-1}H, \qquad \qquad D = \Delta (\widehat{\Sigma }-HH^\top ), \end{aligned}$$
  which are the ML Equations (2.7) and (2.8). If (*H*, *D*) is a solution to these equation also (*HU*, *D*) is a solution, for any unitary matrix $U \in {\mathbb {R}}^{k \times k}$.
- Limit points $({\mathcal {H}},D)$ satisfy
  $$\begin{aligned} {\mathcal {H}}=\widehat{\Sigma }({\mathcal {H}}+D)^{-1}{\mathcal {H}}, \qquad \qquad D=\Delta (\widehat{\Sigma }-{\mathcal {H}}). \end{aligned}$$

#### *Proof*

- This follows from Remark 3.8 and the construction of the algorithm as a combination of the two partial minimizations.
- This similarly follows from Remark 3.8.
- Use the identity $I-H_t^\top (H_tH_t^\top + D_t)^{-1}H_t=(I+H_t^\top D_t^{-1}H_t)^{-1}$ and $\widehat{\Sigma }$ non-negative definite.
- In this case, Equation (4.1) shows that $R_t=I$ and substituting this into the update equations yields the conclusion.
- As matter of fact, we can express the decrease as a sum of two I-divergences, since the algorithm is the superposition of the two partial minimization problems. The results follow from a concatenation of Proposition 3.5 and Proposition 3.7.
- Assume that all variables converge. Then, from (4.3), it follows that Equation (2.9) holds in the limit. This gives the first of the desired relations, the rest is trivial.
- This follows by inserting the result of (f.).

$$\square $$

In part (f) of Proposition 4.4, we made the assumption that the limit points (*H*, *D*) are interior points. This does not always hold true as it may happen that *D* contains zeros on the diagonal. See also Section 6.2.

#### *Remark 4.5*

Assertions (b) and (c) of Proposition 4.4 agree with the recent results of Adachi (2013) (Lemma 1 and Theorem 1) for the closely related EM algorithm with a strictly positive definite empirical covariance matrix $\widehat{\Sigma }$. We note that the assertions (b) and (c) are automatic consequences of our setup, they follow from the casting of the problem as a double divergence minimization problem. Indeed, the solutions to the ensuing partial minimization problems are automatically strictly positive definite matrices, as otherwise the minimum divergences would be infinite, which is impossible.

## Comparison with the EM Algorithm

In Rubin and Thayer (1982), a version of the EM algorithm (see Dempster, Laird, & Rubin, 1977) has been put forward in the context of estimation for FA models. This algorithm is as follows, with $R_t$ as in (4.1).

### **Algorithm 5.1**

(EM)

$$\begin{aligned} H_{t+1}&= \widehat{\Sigma }(H_t H_t^\top + D_t)^{-1}H_t R_t^{-1} \\ D_{t+1}&= \Delta (\widehat{\Sigma }- H_{t+1} R_t H_{t+1}^\top ). \end{aligned}$$

The EM Algorithm 5.1 differs in both equations from our AML Algorithm 4.1. It is well known that EM algorithms can be derived as alternating minimizations, see Csiszár and Tusnády (1984), it is therefore interesting to investigate how Algorithm 5.1 can be derived within our framework. Thereto one considers the first partial minimization problem together with the *constrained* second partial minimization Problem 3.9, the constraint being $Q=Q_0$, for some $Q_0$. Later on we will see that the particular choice of $Q_0$, as long as it is invertible, is irrelevant. The concatenation of these two problems results in the EM Algorithm 5.1, as is detailed below.

Starting at $(H_t,D_t,Q_0)$, one performs the first partial minimization that results in the matrix

$$\begin{aligned} \begin{pmatrix} \widehat{\Sigma } &{} \widehat{\Sigma }(H_tH_t^\top +D_t)^{-1}H_tQ_0 \\ Q_0^\top H_t^\top (H_tH_t^\top +D_t)^{-1}\widehat{\Sigma } &{} Q_0^\top R_t Q_0 \end{pmatrix}. \end{aligned}$$

Performing now the *constrained* second minimization, according to the results of Proposition 3.10, one obtains

5.1 $$\begin{aligned} H_{t+1}&= \widehat{\Sigma }(H_tH_t^\top +D_t)^{-1}H_t R_t^{-1} \end{aligned}$$

5.2 $$\begin{aligned} D_{t+1}&= \Delta \big (\widehat{\Sigma }-\widehat{\Sigma }(H_tH_t^\top +D_t)^{-1}H_t R_t ^{-1} H_t^\top (H_tH_t^\top +D_t)^{-1}\widehat{\Sigma }\big ). \end{aligned}$$

Substitution of (5.1) into (5.2) yields

5.3 $$\begin{aligned} D_{t+1}=\Delta (\widehat{\Sigma }-H_{t+1}R_tH_{t+1}^\top ). \end{aligned}$$

One sees that the matrix $Q_0$ does not appear in the recursion, just as the matrices $Q_t$ do not occur in Algorithm 4.1, but we lost the second optimality property in the construction of Algorithm 4.1, due to the imposed constraint $Q=Q_0$. Moreover, the EM algorithm does not enjoy the diagonal preservation property mentioned in Remark 4.2 for Algorithm 4.1, due to the presence of $R_t$ in Equation (5.3).

Summarizing, both Algorithms 4.1 and 5.1 are the result of two partial minimization problems. The latter algorithm differs from ours in that the second partial minimization is *constrained*. In view of the extra cost incurred by the suboptimal constrained minimization, see Equation (3.9), our Algorithm 4.1 yields a better performance. We will illustrate these considerations by some numerical examples in Section 7.

## Singular *D*

It has been known for a long time, see e.g., Jöreskog (1967), that numerical solutions to the ML Equations (2.7), (2.8) often produce a nearly singular matrix *D*. This motivates the analysis of the minimization Problem 2.1 when *D* is *constrained*, at the outset, to be singular (Section 6.1), and the investigation of its consequences for the minimization algorithm of Proposition 4.3 (Section 6.2).

### Approximation with Singular *D*

In this section, we consider the approximation Problem 2.1 under the constraint $D_2=0$ where

6.1 $$\begin{aligned} D=\begin{pmatrix} D_1 &{} 0 \\ 0 &{} D_2 \end{pmatrix}=\begin{pmatrix} \widetilde{D} &{} 0 \\ 0 &{} 0 \end{pmatrix}, \end{aligned}$$

with $D_1=\widetilde{D}>0$ of size $n_1$ and $D_2=0$ of size $n_2$. The constrained minimization problem can be formulated as follows.

#### **Problem 6.1**

Given $\widehat{\Sigma }>0$ of size $n\times n$ and integers $n_2$ and *k*, with $n_2 \le k < n$, minimize

$$\begin{aligned} {\mathcal {I}}(\widehat{\Sigma } || HH^\top +D), \end{aligned}$$

over (*H*, *D*) with *D* satisfying (6.1).

#### *Remark 6.2*

Alternatively, in Jöreskog (1967), the solution of the likelihood Equations (2.8) and (2.9) has been investigated under zero constraints on *D*. In this section, we work directly on the objective function of Problem 6.1.

To reduce the complexity of Problem 6.1 we will now decompose the objective function, choosing a convenient representation of the matrix $$H=\begin{pmatrix} H_1 \\ H_2 \end{pmatrix}$$, where $H_i$ has $n_i$ rows. Inspired by the parametrization in Jöreskog (1967) we make the following observation. Given any orthogonal matrix *Q*, define $H' = HQ$, then clearly $H'H'^\top + D = HH^\top +D$. Let $H_{2}=U(0\,\, \Lambda )V^{\top }$ be the singular value decomposition of $H_{2}$, with $\Lambda$ a positive definite diagonal matrix of size $n_{2}\times n_{2}$, and *U* and *V* orthogonal of sizes $n_{2}\times n_{2}$ and $k\times k$, respectively. Let

$$\begin{aligned} H'= HV \end{aligned}$$

The blocks of $H'$ are $H_{1}' =H_{1}V$ and $H_{2}'=(H_{21}'\, H_{22}'):=(0\quad U\Lambda )$, with $H_{21}'\in {\mathbb {R}}^{(k-n_2)\times n_2}$ and $H'_{22}\in {\mathbb {R}}^{n_2\times n_2}$. Hence, without loss of generality, in the remainder of this section we assume that

6.2 $$\begin{aligned} H=\begin{pmatrix} H_1 \\ H_2 \end{pmatrix} = \begin{pmatrix} H_{11} &{} H_{12} \\ 0 &{} H_{22} \end{pmatrix}, \qquad H_{22} \,\, \hbox {invertible}. \end{aligned}$$

Finally, let

$$\begin{aligned} K=\widehat{\Sigma }_{12}\widehat{\Sigma }_{22}^{-1}-H_1H_2^\top (H_2H_2^\top )^{-1}, \end{aligned}$$

which, under (6.2), is equivalent to

$$\begin{aligned} K=\widehat{\Sigma }_{12}\widehat{\Sigma }_{22}^{-1}-H_{12}H_{22}^{-1}. \end{aligned}$$

Here is the announced decomposition of the objective function.

#### **Lemma 6.3**

Let *D* be as in Equation (6.1). The following I-divergence decomposition holds.

6.3 $$\begin{aligned} {\mathcal {I}}(\widehat{\Sigma }||HH^{\top }+D) =&\, {\mathcal {I}}(\widetilde{\Sigma }_{11}||H_{11}H_{11}^{\top }+ \widetilde{D})+{\mathcal {I}}(\widehat{\Sigma }_{22}||H_{22}H_{22}^{\top })\nonumber \\&+\frac{1}{2}\text{ tr }\big (\widehat{\Sigma }_{22}K^\top (H_{11}H_{11}^{\top } +\widetilde{D})^{-1}K\big ). \end{aligned}$$

#### *Proof*

The proof follows from Lemma 9.1. $\square$

We are now ready to characterize the solution of Problem 6.1. Observe first that the second and third terms on the right-hand side of (6.3) are non-negative and can be made zero. To this end, it is enough to select $H_{22}$ such that $H_{22}H_{22}^{\top }=\widehat{\Sigma }_{22}$ and then $H_{12}=\widehat{\Sigma }_{12}\widehat{\Sigma }_{22}^{-1}H_{22}$. The remaining blocks, $H_{11}$ and $\widetilde{D}$, are determined minimizing the first term. We have thus proved the following

#### **Proposition 6.4**

Any pair (*H*, *D*), as in (6.1) and (6.2), solving Problem 6.1 satisfies

(i) $${\mathcal {I}}(\widetilde{\Sigma }_{11}||H_{11}H_{11}^{\top }+\widetilde{D})$$ is minimized,
(ii) $${\mathcal {I}}(\widehat{\Sigma }||HH^\top + D) = {\mathcal {I}}(\widetilde{\Sigma }_{11}||H_{11}H_{11}^{\top }+\widetilde{D})$$,
(iii) $$H_{22}H_{22}^{\top } = \widehat{\Sigma }_{22}$$,
(iv) $$H_{12} = \widehat{\Sigma }_{12}\widehat{\Sigma }_{22}^{-1}H_{22}$$.

#### *Remark 6.5*

In the special case $n_{2}=k$, the matrices $H_{11}$ and $H_{21}$ are empty, $H_{12}=H_{1}$, and $H_{22}=H_{2}$. From Proposition 6.4, at the minimum, $H_{2}H_{2}^{\top }=\widehat{\Sigma }_{22}$, $H_{1}H_2^\top =\widehat{\Sigma }_{12}$, and $\widetilde{D}$ minimizes ${\mathcal {I}}(\widetilde{\Sigma }_{11}||\widetilde{D})$. The latter problem has solution $\widetilde{D}=\Delta (\widetilde{\Sigma }_{11})$. It is remarkable that in this case the minimization problem has an *explicit* solution.

### Algorithm When a Part of *D* has Zero Diagonal

In Section 6.1, we have posed the minimization problem under the additional constraint that the matrix *D* contains a number of zeros on the diagonal. In the present section, we investigate how this constraint affects the alternating minimization algorithm. For simplicity, we give a detailed account of this, only using the recursion (4.7) for ${\mathcal {H}}_t$. Initialize the algorithm at $(H_0, D_0)$ with

6.4 $$\begin{aligned} D_0 = \begin{pmatrix} \widetilde{D}_0 &{} 0 \\ 0 &{} 0 \end{pmatrix}, \end{aligned}$$

where $$\widetilde{D}_0>0$$, and

6.5 $$\begin{aligned} H_0=\begin{pmatrix} H_{10} \\ H_{20} \end{pmatrix}, \end{aligned}$$

where $H_{20}\in {\mathbb {R}}^{n_2\times k}$ is assumed to have full row rank, so that $n_2\le k$. Clearly, $H_0H_0^\top + D_0$ is invertible. For $H_0$ as in Equation (6.5) put

6.6 $$\begin{aligned} \widetilde{{\mathcal {H}}}_0= H_{10}(I-H_{20}^\top (H_{20}H_{20}^\top )^{-1}H_{20})H_{10}^\top . \end{aligned}$$

#### **Proposition 6.6**

Consider the update Equation (4.7). The upper left block ${\mathcal {H}}^{11}_t$ of ${\mathcal {H}}_t$ can be computed running a recursion for $\widetilde{{\mathcal {H}}}_t := {\mathcal {H}}^{11}_t-\widehat{\Sigma }_{12}\widehat{\Sigma }_{22}^{-1}\widehat{\Sigma }_{21}$, with initial condition $\widetilde{{\mathcal {H}}}_0$,

$$\begin{aligned} \widetilde{{\mathcal {H}}}_{t+1}= \widetilde{\Sigma }_{11}(\widetilde{{\mathcal {H}}}_t +\widetilde{D}_t)^{-1}\widetilde{{\mathcal {H}}}_t(\widetilde{D}_t+ \widetilde{\Sigma }_{11}(\widetilde{{\mathcal {H}}}_t +\widetilde{D}_t)^{-1}\widetilde{{\mathcal {H}}}_t)^{-1}\widetilde{\Sigma }_{11}, \end{aligned}$$

whereas the blocks on the border of ${\mathcal {H}}_t$ remain constant. The iterates for $D_t$ all have a lower right block of zeros, while the upper left $n_1 \times n_1$ block $\widetilde{D}_t$ satisfies

$$\begin{aligned} \widetilde{D}_{t} = \Delta (\widetilde{\Sigma }_{11} - \widetilde{{\mathcal {H}}}_{t}). \end{aligned}$$

Limit points $(\widetilde{{\mathcal {H}}},\widetilde{D})$ with $\widetilde{D}>0$ satisfy $\widetilde{{\mathcal {H}}}=\widetilde{\Sigma }_{11}(\widetilde{{\mathcal {H}}}+ \widetilde{D})^{-1}\widetilde{{\mathcal {H}}}$, $\widetilde{D}=\Delta (\widetilde{\Sigma }_{11}-\widetilde{{\mathcal {H}}})$.

#### *Proof*

See Appendix 2. $$\square $$

Note that the recursions of Proposition 6.6 are exactly those that follow from the optimization Problem 6.1. Comparison with (4.7) shows that, while the algorithm for the unconstrained case updates ${\mathcal {H}}_t$ of size $n\times n$, now one needs to update $\widetilde{{\mathcal {H}}}_t$ which is of smaller size $n_1\times n_1$.

In the special case $n_2=k$, the matrix $\widetilde{{\mathcal {H}}}_0$ of (6.6) is equal to zero. Therefore, $\widetilde{{\mathcal {H}}}^{11}_1 =\widehat{\Sigma }_{12}\widehat{\Sigma }_{22}^{-1}\widehat{\Sigma }_{21}$ which proves the following

#### **Corollary 6.7**

Let the initial value $D_0$ be as in Equation (6.4) with $n_2=k$. Then for any initial value ${\mathcal {H}}_0$, the algorithm converges *in one step* and one has that the first iterates $D_1$ and ${\mathcal {H}}_1$, which are equal to the terminal values, are given by

$$\begin{aligned} D_1&= \begin{pmatrix} \Delta (\widetilde{\Sigma }_{11}) &{} 0 \\ 0 &{} 0 \end{pmatrix} \\ {\mathcal {H}}_1&= \begin{pmatrix} \widehat{\Sigma }_{12}\widehat{\Sigma }_{22}^{-1}\widehat{\Sigma }_{21} &{} \widehat{\Sigma }_{12} \\ \widehat{\Sigma }_{21} &{} \widehat{\Sigma }_{22} \end{pmatrix}. \end{aligned}$$

It is remarkable that in this case the algorithm reaches *in one step*, the optimal values are computed explicitly in Remark 6.5.

## Numerical Comparisons with Other Algorithms

We briefly investigate the numerical performance of our AML Algorithm 4.1, and compare it against the performance of other algorithms. The natural competitor of AML is the EM Algorithm 5.1. After the publication of Rubin and Thayer (1982), the EM algorithm has evolved into a cohort of improved alternatives (Liu & Rubin, 1994, 1998, and more recently by Zhao et al., 2008), basically differing from the original EM on numerical implementation aspects. Most notably, in the ECME variant (Liu & Rubin, 1998), $H_t$ is updated as in the EM algorithm, but $D_{t}$ is updated by direct maximization of the likelihood (equivalently minimization of the I-divergence) with respect to *D*, keeping *H* fixed at the value $H_{t+1}$. This step cannot be done analytically, and is realized taking a few Newton–Raphson iterations, and Liu and Rubin (1998) suggests that one or two iterations are usually sufficient. The resulting $D_{t+1}$ does not necessarily increase the likelihood with respect to $D_t$; therefore, a check has to be performed, and possibly the iteration has to be repeated adjusting its size. The rationale behind ECME is that the advantage in speed afforded by the direct (along the *D* parameter) maximization of the likelihood outweighs the drawback of having to check each iteration for actual improvement. We have derived, in the same spirit, a variant of AML retaining the $H_t$ update Equation (4.5) and replacing the $D_t$ update Equation (4.6) with the same Newton–Raphson iterations as in ECME. We named the resulting algorithm ACML. All numerical experiments in this section should be read as comparisons between the performances of AML and ACML versus EM and ECME.

### Findings

To run the numerical comparisons, we have selected from the published literature five examples of correlation matrices, some of which are well known for being problematic for FA modeling. We have also constructed a sixth data set as an *exact* FA covariance $\widehat{\Sigma } = HH^\top +D$, selecting randomly the entries of *H* and *D*, see below. For each of the six data sets we ran the four algorithms in parallel (sharing the same initial conditions) several times, changing the initial conditions at each run. The figures at the end of the paper are plots of the I-divergence vs. iterations and have been selected to show the typical behaviors of the four algorithms for each data set. The data sets are the following correlation matrices $\widehat{\Sigma }$ of size $n\times n$.

**Fig. 1:**
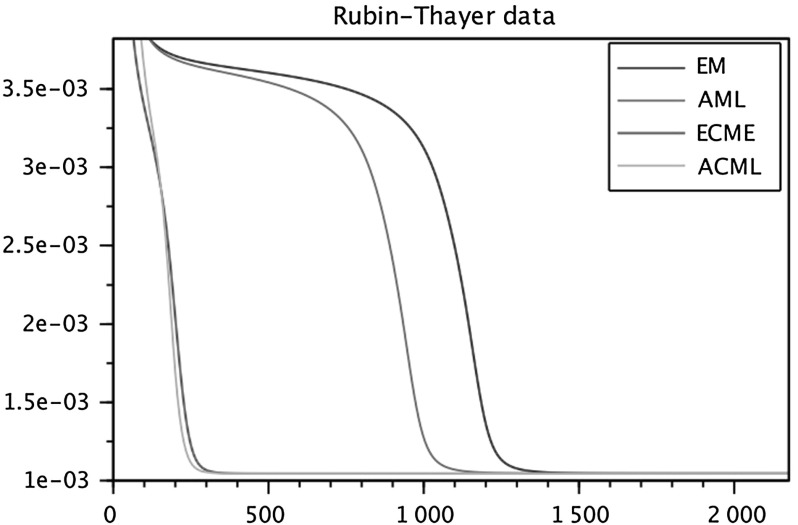
Rubin–Thayer.

**Fig. 2:**
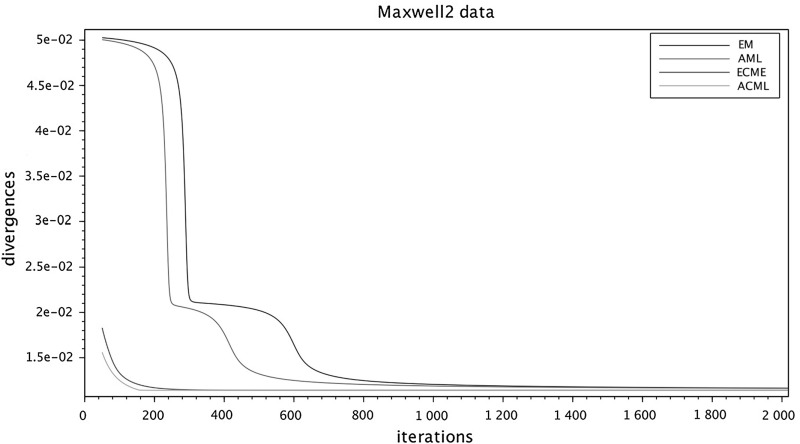
Maxwell.

**Fig. 3:**
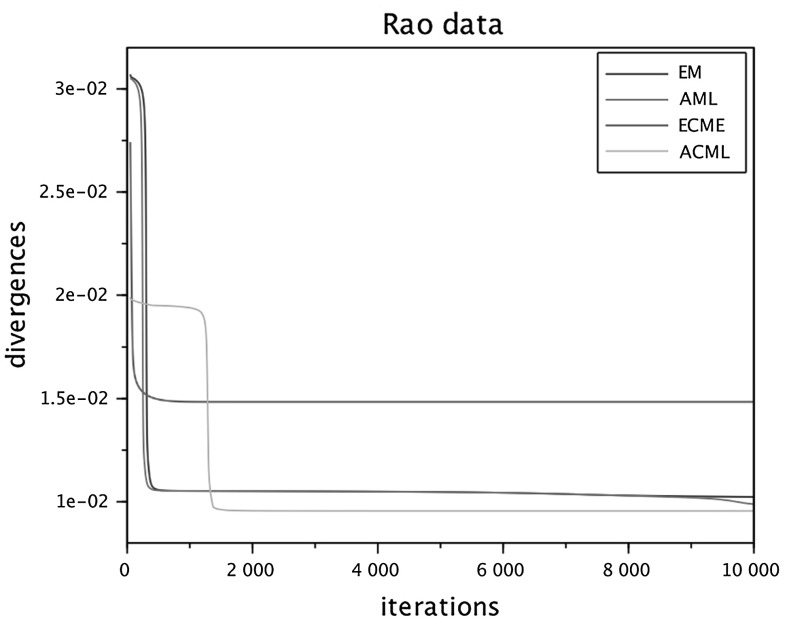
Rao.

**Fig. 4:**
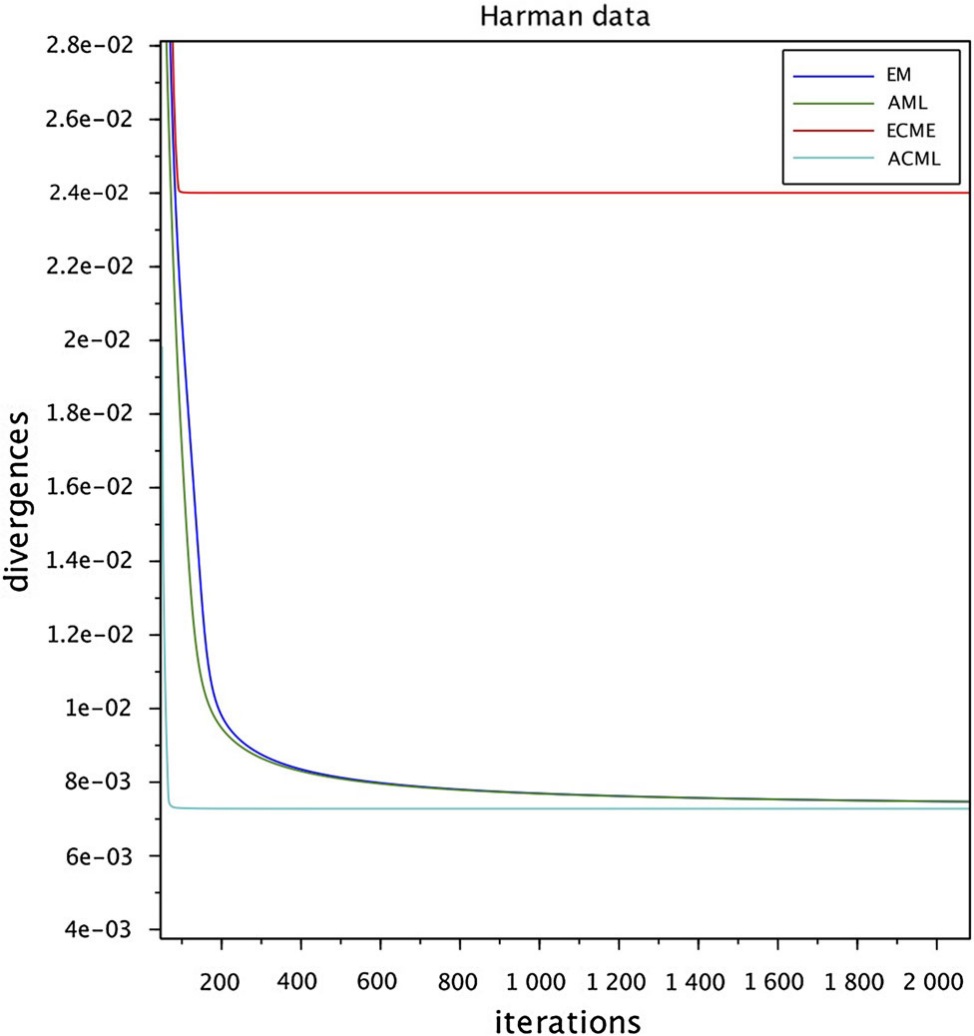
Harman.

**Fig. 5:**
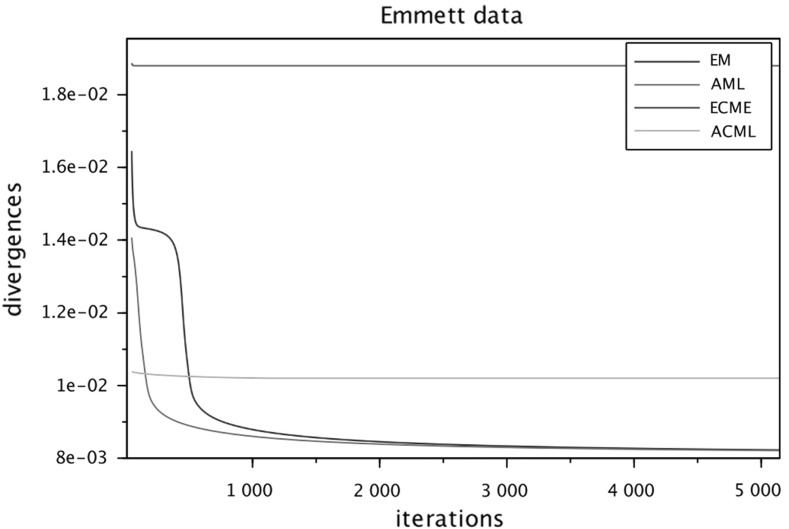
Emmett.

**Fig. 6:**
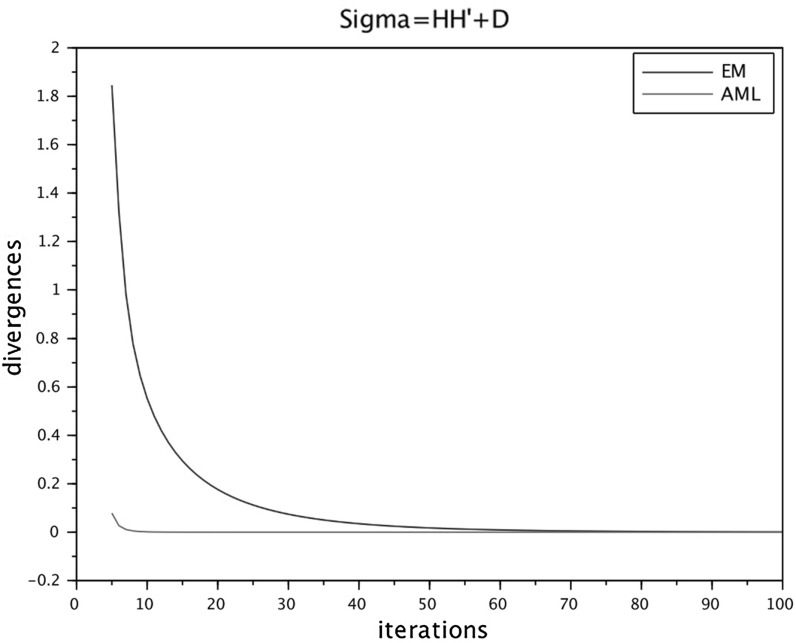
True FA model.

- Figure 1: Rubin–Thayer, $n=9$, taken from Rubin and Thayer (1982).
- Figure 2: Maxwell, $n=9$, Table 4 in Maxwell (1961), also analyzed as data set 2 in Jöreskog (1967).
- Figure 3: Rao, $n=9$, taken from Rao (1955).
- Figure 4: Harman, $n=8$, Table 5.3 in Harman (1967).
- Figure 5: Emmett, $n=9$, Table I in Emmett (1949), also analyzed as data set 1 in Jöreskog (1967).
- Figure 6: The data set is a randomly generated covariance of the standard FA model type, i.e., $\widehat{\Sigma } = HH^\top +\gamma D$, with $n=20$. The elements of $H\in \mathbb {R}^{20\times 4}$ and of $D\in \mathbb {R}^{20\times 20}$ are samples of a uniform on $[1, \, 10]$. For the choice of $\gamma \in \mathbb {R}_+$ see below under (c2).

In all numerical experiments, the number of factors has been fixed, equal to $k=4$. Initially it was found that, for a number of runs with different data sets and initial conditions, the ECME algorithm produced negative values for the diagonal matrices $D_t$ caused by a routine application of the Newton–Raphson (NR) algorithm. The NR routine has afterward been improved, implementing the restricted step version of the NR algorithm for both ECME and ACML. In all ECME and ACML experiments, we have consistently taken 2 steps of the NR algorithm at each iteration. To present the findings, we have grouped the data sets into three groups (a.), (b.), and (c.), within which we observed similar behaviors. Different behaviors are ranked according to their limit divergence and speed of convergence, with priority given to the former.

(a1): **Rubin–Thayer data** (Figure 1). The graphs of the EM/ECME pair are very similar to those of Liu and Rubin (1998) and we observe that the AML/ACML pair outperforms EM/ECME. The typical ranking for this data set was ACML best, followed by ECME, AML, EM in that order. In a few occasions we observed AML best, followed by EM, ACML, and ECME. The ECME was the most sensitive to initial conditions.
(a2): **Maxwell data** (Figure 2). The typical ranking for this data set is as above, ACML, ECME, AML, EM, in decreasing order. For both ECME and ACML we have been able to reproduce Table 5 of Jöreskog for the elements of the *D*-matrix, and also identified the eighth element of the *D*-matrix as $D_8=0$. In a few occasions ACML and ECME displayed very close behaviors, significantly outperforming AML and EM whose behaviors were also close to each other.
(b1): **Rao data** (Figure 3). The typical ranking for the data set was ACML, AML, EM, and ECME. Sometimes it took more than 1500 iterations before a significant drop in the divergence of the best performing algorithm could be seen. The $D_1$ should be estimated close to zero (Jennrich & Robinson, 1969), which was usually the case for ACML, for AML and EM with slower convergence. ECME displayed different behaviors (sometimes very good), depending on the initial conditions.
(b2): **Harman data** (Figure 4). The typical ranking for the data set was as above, ACML, AML, EM, ECME. In our runs, ACML performed consistently best, whereas ECME consistently exhibited much larger divergences. For this data set, $D_2$ is known to be zero (Jennrich & Robinson, 1969). All runs of the ACML have quickly produced $D_2=0$, sometimes ECME too, although large deviations have been seen as well. AML and ME exhibited much slower convergence, often 5000 iterations were not enough.
(c1): **Emmett data** (Figure 5). The behavior of the four algorithms for this data set is exceptional. Very often AML and EM gave faster and better (i.e., to smaller values) convergence than ACML and ECME.
(c2): **True FA covariance matrix** (Figure 6). Since $\widehat{\Sigma } = HH^\top +\gamma D$, where $H\in \mathbb {R}^{20\times 4}$ and the selected number of factors is $k=4$, this is a *perfect* modeling situation, with vanishing theoretical minimum divergence. The AML is the best performer, reaching null divergence extremely fast, while the ranking of the other algorithms is sensitive to the value of $\gamma$. Figure 6, for $\gamma =10$, shows AML and EM. The pair ACML and ECME has a much worse behavior, which cannot be plotted on the same graph. For $\gamma =0.1$, the ranking of behaviors is different. AML is still the best, immediately followed by ACML, whereas ECME and EM behave erratically and do not converge to zero. We omitted the figure.

## Conclusions

Given a positive definite covariance matrix $\widehat{\Sigma }$, which may be an empirical covariance, of dimension *n*, we considered the problem of approximating it with a covariance of the form $HH^\top +D$, where *H* has a prescribed low number columns and $D>0$ is diagonal. We have chosen to gauge the quality of the approximation by the I-divergence between the zero mean normal laws with covariances $\widehat{\Sigma }$ and $HH^\top +D$, respectively. By lifting the minimization problem into a larger space, we have been able to develop an optimal procedure from first principles to determine a pair (*H*, *D*) that minimizes the I-divergence. As consequence, the procedure also yields an iterative alternating minimization algorithm (AML) à la Csiszár–Tusnády. As it turns out, the proper choice of the enlarged space is crucial for optimization. We have obtained a number of theoretical results that are of independent interest. The convergence of the algorithm has also been studied, with special attention given to the case where *D* is singular. The theoretical properties of the AML have been compared to those of the popular EM algorithm for exploratory factor analysis. Inspired by the ECME (a Newton–Raphson variation on EM), we also developed a similar variant of AML, called ACML, and in a few numerical experiments we compared the performances of the four algorithms. We have seen that usually the ACML algorithm performed best, in particular, better than ECME. In some specific experiments, AML was best, and always outperforming the EM algorithm.

## Appendix 1: Decompositions of the I-Divergence

Recall that, for given probability measures ${\mathbb {P}}_1$ and ${\mathbb {P}}_2$, defined on the same measurable space, and such that ${\mathbb {P}}_1\ll {\mathbb {P}}_2$, the I-divergence is defined as

9.1 $$\begin{aligned} {\mathcal {I}}({\mathbb {P}}_1||{\mathbb {P}}_2)=\mathbb {E}_{{\mathbb {P}}_1}\log \frac{\mathrm{d}{\mathbb {P}}_1}{\mathrm{d}{\mathbb {P}}_2}, \end{aligned}$$

where $\frac{\mathrm{d}{\mathbb {P}}_1}{\mathrm{d}{\mathbb {P}}_2}$ denotes the Radon-Nikodym derivative of ${\mathbb {P}}_1$ w.r.t. ${\mathbb {P}}_2$. If ${\mathbb {P}}_1$ and ${\mathbb {P}}_2$ are distributions on $\mathbb {R}^m$ of a random vector *X* with corresponding densities $f_1$ and $f_2$ that are everywhere positive, the Radon-Nikodym derivative $\frac{\mathrm{d}{\mathbb {P}}_1}{\mathrm{d}{\mathbb {P}}_2}$ becomes the likelihood ratio $\frac{f_1(X)}{f_2(X)}$ and (9.1) reduces to the integral

9.2 $$\begin{aligned} {\mathcal {I}}({\mathbb {P}}_1||{\mathbb {P}}_2) =\int _{\mathbb {R}^m} f_1(x)\log \frac{f_1(x)}{f_2(x)}\,\mathrm{d}x. \end{aligned}$$

We note that all subsequent expressions for the I-divergence have similar counterparts in terms of densities. In this section we derive a number of decomposition results for the I-divergence between two probability measures. Similar results are derived in Cramer (2000), see also Finesso and Spreij (2006) for the discrete case. These decompositions yield the core arguments for the proofs of the propositions in Sections 3.1 and 6.1.

### **Lemma 9.1**

Let ${\mathbb {P}}_{XY}$ and ${\mathbb {Q}}_{XY}$ be given probability distributions of a Euclidean random vector (*X*, *Y*) and denote by ${\mathbb {P}}_{X|Y}$ and ${\mathbb {Q}}_{X|Y}$ the corresponding regular conditional distributions of *X* given *Y*. Assume that ${\mathbb {P}}_{XY} \ll {\mathbb {Q}}_{XY}$. Then

9.3 $$\begin{aligned} {\mathcal {I}}({\mathbb {P}}_{XY}||{\mathbb {Q}}_{XY})={\mathcal {I}}({\mathbb {P}}_Y||{\mathbb {Q}}_Y) +\mathbb {E}_{{\mathbb {P}}_Y}{\mathcal {I}}({\mathbb {P}}_{X|Y}||{\mathbb {Q}}_{X|Y}). \end{aligned}$$

### *Proof*

It is easy to see that we also have ${\mathbb {P}}_Y \ll {\mathbb {Q}}_Y$. Moreover, we also have absolute continuity of the conditional laws, in the sense that if 0 is a version of the conditional probability ${\mathbb {Q}}(X\in B|Y)$, then it is also a version of ${\mathbb {P}}(X\in B|Y)$. One can show that a conditional version of the Radon-Nikodym theorem applies and that a conditional Radon-Nikodym derivative $\frac{\mathrm{d}{\mathbb {P}}_{X|Y}}{\mathrm{d}{\mathbb {Q}}_{X|Y}}$ exists ${\mathbb {Q}}_Y$ almost surely. Moreover, one has the ${\mathbb {Q}}_{XY}$ as factorization

$$\begin{aligned} \frac{\mathrm{d}{\mathbb {P}}_{XY}}{\mathrm{d}{\mathbb {Q}}_{XY}}=\frac{\mathrm{d}{\mathbb {P}}_{X|Y}}{\mathrm{d}{\mathbb {Q}}_{X|Y}}\frac{\mathrm{d}{\mathbb {P}}_{Y}}{\mathrm{d}{\mathbb {Q}}_{Y}}. \end{aligned}$$

Taking logarithms on both sides, and expectation under ${\mathbb {P}}_{XY}$ yields

$$\begin{aligned} \mathbb {E}_{{\mathbb {P}}_{XY}}\log \frac{\mathrm{d}{\mathbb {P}}_{XY}}{\mathrm{d}{\mathbb {Q}}_{XY}} =\mathbb {E}_{{\mathbb {P}}_{XY}}\log \frac{\mathrm{d}{\mathbb {P}}_{X|Y}}{\mathrm{d}{\mathbb {Q}}_{X|Y}} +\mathbb {E}_{{\mathbb {P}}_{XY}}\log \frac{\mathrm{d}{\mathbb {P}}_{Y}}{\mathrm{d}{\mathbb {Q}}_{Y}}. \end{aligned}$$

Writing the first term on the right-hand side as $\mathbb {E}_{{\mathbb {P}}_{XY}}\{\mathbb {E}_{{\mathbb {P}}_{XY}}[\log \frac{\mathrm{d}{\mathbb {P}}_{X|Y}}{\mathrm{d}{\mathbb {Q}}_{X|Y}}|Y]\}$, we obtain $\mathbb {E}_{{\mathbb {P}}_{Y}}\{\mathbb {E}_{{\mathbb {P}}_{X|Y}}[\log \frac{\mathrm{d}{\mathbb {P}}_{X|Y}}{\mathrm{d}{\mathbb {Q}}_{X|Y}}|Y]\}$. The result follows. $\square$

The decomposition of Lemma 9.1 is useful when solving I-divergence minimization problems with marginal constraints, like the one considered below.

### **Proposition 9.2**

Let ${\mathbb {Q}}_{XY}$ and ${\mathbb {P}}^0_Y$ be given probability distributions of a Euclidean random vector (*X*, *Y*), and of its subvector *Y*, respectively. Consider the I-divergence minimization problem

$$\begin{aligned} \min _{{\mathbb {P}}_{XY} \in \mathcal {P}} \, {\mathcal {I}}({\mathbb {P}}_{XY}||{\mathbb {Q}}_{XY}), \end{aligned}$$

where

$$\begin{aligned} \mathcal {P} := \left\{ {\mathbb {P}}_{XY} \, \Big | \, \int {\mathbb {P}}_{XY}(dx, Y) = {\mathbb {P}}^0_Y \right\} . \end{aligned}$$

If the marginal ${\mathbb {P}}^0_Y \ll {\mathbb {Q}}^0_Y$, then the I-divergence is minimized by ${\mathbb {P}}^*_{XY}$ specified by the Radon-Nikodym derivative

9.4 $$\begin{aligned} \frac{\mathrm{d}{\mathbb {P}}^*_{XY}}{\mathrm{d}{\mathbb {Q}}_{XY}}=\frac{\mathrm{d}{\mathbb {P}}^0_Y}{\mathrm{d}{\mathbb {Q}}_Y}. \end{aligned}$$

Moreover, the Pythagorean rule holds i.e.  for any other distribution ${\mathbb {P}}\in \mathcal {P}$,

9.5 $$\begin{aligned} {\mathcal {I}}({\mathbb {P}}_{XY}||{\mathbb {Q}}_{XY})={\mathcal {I}}({\mathbb {P}}_{XY}||{\mathbb {P}}^*_{XY})+{\mathcal {I}}({\mathbb {P}}^*_{XY}||{\mathbb {Q}}_{XY}), \end{aligned}$$

and one also has

9.6 $$\begin{aligned} {\mathcal {I}}({\mathbb {P}}^*_{XY}||{\mathbb {Q}}_{XY})={\mathcal {I}}({\mathbb {P}}^0_Y||{\mathbb {Q}}_Y). \end{aligned}$$

### *Proof*

The starting point is Equation (9.3), which now takes the form

9.7 $$\begin{aligned} {\mathcal {I}}({\mathbb {P}}_{XY}||{\mathbb {Q}}_{XY})={\mathcal {I}}({\mathbb {P}}^0_Y||{\mathbb {Q}}_Y) +\mathbb {E}_{{\mathbb {P}}_Y}{\mathcal {I}}({\mathbb {P}}_{X|Y}||{\mathbb {Q}}_{X|Y}). \end{aligned}$$

Since the first term on the right-hand side is fixed, the minimizing ${\mathbb {P}}^*_{XY}$ must satisfy ${\mathbb {P}}^*_{X|Y}={\mathbb {Q}}_{X|Y}$. It follows that ${\mathbb {P}}^*_{XY}={\mathbb {P}}^*_{X|Y}{\mathbb {P}}^0_Y={\mathbb {Q}}_{X|Y}{\mathbb {P}}^0_Y$, thus verifying (9.4) and (9.6). We finally show that (9.5) holds.

$$\begin{aligned} {\mathcal {I}}({\mathbb {P}}_{XY}||{\mathbb {Q}}_{XY})&=\mathbb {E}_{{\mathbb {P}}_{XY}} \log \frac{\mathrm{d}{\mathbb {P}}_{XY}}{\mathrm{d}{\mathbb {P}}^*_{XY}}+\mathbb {E}_{{\mathbb {P}}_{XY}} \log \frac{\mathrm{d}{\mathbb {P}}^*_{XY}}{\mathrm{d}{\mathbb {Q}}_{XY}} \\&= {\mathcal {I}}({\mathbb {P}}_{XY}||{\mathbb {P}}^*_{XY}) + \mathbb {E}_{{\mathbb {P}}_Y}\log \frac{\mathrm{d}{\mathbb {P}}^0_Y}{\mathrm{d}{\mathbb {Q}}_Y} \\&= {\mathcal {I}}({\mathbb {P}}_{XY}||{\mathbb {P}}^*_{XY}) + \mathbb {E}_{{\mathbb {P}}^0_Y}\log \frac{\mathrm{d}{\mathbb {P}}^0_Y}{\mathrm{d}{\mathbb {Q}}_Y}, \end{aligned}$$

where we used that any ${\mathbb {P}}_{XY} \in \mathcal {P}$ has *Y*-marginal distribution ${\mathbb {P}}^0_Y$. $\square$

The results above can be extended to the case where the random vector $(X, Y):=(X, Y_1, \dots Y_m)$, i.e., *Y* consists of *m* random subvectors $Y_i$. For any probability distribution ${\mathbb {P}}_{XY}$ on (*X*, *Y*), consider the conditional distributions ${\mathbb {P}}_{Y_i|X}$ and define the probability distribution $\widetilde{{\mathbb {P}}}_{XY}$ on (*X*, *Y*):

$$\begin{aligned} \widetilde{{\mathbb {P}}}_{XY} = \prod _i {\mathbb {P}}_{Y_i|X} {\mathbb {P}}_X. \end{aligned}$$

Note that, under $\widetilde{{\mathbb {P}}}_{XY}$, the $Y_i$ are conditionally independent given *X*. The following lemma sharpens Lemma 9.1.

### **Lemma 9.3**

Let ${\mathbb {P}}_{XY}$ and ${\mathbb {Q}}_{XY}$ be given probability distributions of a Euclidean random vector $(X,Y):=(X,Y_1,\dots Y_m)$. Assume that ${\mathbb {P}}_{XY} \ll {\mathbb {Q}}_{XY}$ and that, under ${\mathbb {Q}}_{XY}$, the subvectors $Y_i$ of *Y* are conditionally independent given *X*, then

$$\begin{aligned} {\mathcal {I}}({\mathbb {P}}_{XY}||{\mathbb {Q}}_{XY})={\mathcal {I}}({\mathbb {P}}_{XY}||\widetilde{{\mathbb {P}}}_{XY}) +\sum _i\mathbb {E}_{{\mathbb {P}}_X}{\mathcal {I}}({\mathbb {P}}_{Y_i|X}||{\mathbb {Q}}_{Y_i|X}) + {\mathcal {I}}({\mathbb {P}}_X||{\mathbb {Q}}_X). \end{aligned}$$

### *Proof*

The proof runs along the same lines as the proof of Lemma 9.1. $\square$

The decomposition of Lemma 9.3 is useful when solving I-divergence minimization problems with conditional independence constraints, like the one considered below.

### **Proposition 9.4**

Let ${\mathbb {P}}_{XY}$ be a given probability distribution of a Euclidean random vector $(X,Y):=(X,Y_1,\dots Y_m)$. Consider the I-divergence minimization problem

$$\begin{aligned} \min _{{\mathbb {Q}}_{XY} \in \mathcal {Q}} \, {\mathcal {I}}({\mathbb {P}}_{XY}||{\mathbb {Q}}_{XY}), \end{aligned}$$

where

$$\begin{aligned} \mathcal {Q} := \left\{ {\mathbb {Q}}_{XY} \,\, \Big | \,\, {\mathbb {Q}}_{Y_1, \dots , Y_m|X} = \prod _i {\mathbb {Q}}_{Y_i|X} \right\} . \end{aligned}$$

If ${\mathbb {P}}_{XY} \ll {\mathbb {Q}}_{XY}$ for some ${\mathbb {Q}}_{XY} \in \mathcal {Q}$ then the I-divergence is minimized by

$$\begin{aligned} {\mathbb {Q}}^*_{XY}=\widetilde{{\mathbb {P}}}_{XY} \end{aligned}$$

Moreover, the Pythagorean rule holds, i.e., for any ${\mathbb {Q}}_{XY} \in \mathcal {Q}$,

$$\begin{aligned} {\mathcal {I}}({\mathbb {P}}_{XY}||{\mathbb {Q}}_{XY})= {\mathcal {I}}({\mathbb {P}}_{XY}||{\mathbb {Q}}^*_{XY}) + {\mathcal {I}}({\mathbb {Q}}^*_{XY}||{\mathbb {Q}}_{XY}). \end{aligned}$$

### *Proof*

From the right-hand side of the identity in Lemma 9.3, we see that the first I-divergence is not involved in the minimization, whereas the other two can be made equal to zero, by selecting ${\mathbb {Q}}_{Y_i|X}={\mathbb {P}}_{Y_i|X}$ and ${\mathbb {Q}}_X={\mathbb {P}}_X$. This shows that the minimizing ${\mathbb {Q}}^*_{XY}$ is equal to $\widetilde{{\mathbb {P}}}_{XY}$. To prove the Pythagorean rule, we first observe that trivially

9.8 $$\begin{aligned} {\mathcal {I}}({\mathbb {P}}_{XY}|{\mathbb {Q}}^*_{XY})={\mathcal {I}}({\mathbb {P}}_{XY}|\widetilde{{\mathbb {P}}}_{XY}). \end{aligned}$$

Next we apply the identity in Lemma 9.3 with ${\mathbb {Q}}^*_{XY}$ replacing ${\mathbb {P}}_{XY}$. In this case the corresponding $\widetilde{{\mathbb {Q}}}^*_{XY}$ obviously equals ${\mathbb {Q}}^*_{XY}$ itself. Hence the identity reads

9.9 $$\begin{aligned} {\mathcal {I}}({\mathbb {Q}}^*_{XY}||{\mathbb {Q}}_{XY})&= \sum _i\mathbb {E}_{{\mathbb {Q}}^*_X}{\mathcal {I}}({\mathbb {Q}}^*_{Y_i|X}||{\mathbb {Q}}_{Y_i|X}) + {\mathcal {I}}({\mathbb {Q}}^*_X ||{\mathbb {Q}}_X) \nonumber \\&= \sum _i\mathbb {E}_{{\mathbb {P}}_X}{\mathcal {I}}({\mathbb {P}}_{Y_i|X}||{\mathbb {Q}}_{Y_i|X}) + {\mathcal {I}}({\mathbb {P}}_X||{\mathbb {Q}}_X), \end{aligned}$$

by definition of ${\mathbb {Q}}^*_{XY}$. Adding up Equations (9.8) and (9.9) gives the result. $\square$

## Appendix 2: Proof of the Technical Results

### *Proof of Proposition 3.5*

(*First partial minimization*). Consider the setup and the notation of Proposition 9.2. Identify ${\mathbb {Q}}$ with the normal $N(0,\Sigma )$, and ${\mathbb {P}}$ with $N(0,\Sigma _0)$. By virtue of (9.4), the optimal ${\mathbb {P}}^*$ is a zero mean normal whose covariance matrix can be computed using the properties of conditional normal distributions. In particular,

$$\begin{aligned} \Sigma ^*_{21} = \mathbb {E}_{{\mathbb {P}}^*}XY^\top&= \mathbb {E}_{{\mathbb {P}}^*}(\mathbb {E}_{{\mathbb {P}}^*}[X|Y]Y^\top )\\&=\mathbb {E}_{{\mathbb {P}}^*}(\mathbb {E}_{\mathbb {Q}}[X|Y]Y^\top )\\&=\mathbb {E}_{{\mathbb {P}}^*}(\Sigma _{21}\Sigma _{11}^{-1}YY^\top ) \\&= \Sigma _{21}\Sigma _{11}^{-1}\mathbb {E}_{{\mathbb {P}}^0}YY^\top \\&=\Sigma _{21}\Sigma _{11}^{-1}\widehat{\Sigma }. \end{aligned}$$

Likewise

$$\begin{aligned} \Sigma ^*_{22} = \Sigma _{22}-\Sigma _{21}\Sigma _{11}^{-1}\Sigma _{12} + \Sigma _{21}\Sigma _{11}^{-1}\widehat{\Sigma }\Sigma _{11}^{-1}\Sigma _{12}. \end{aligned}$$

To prove that $\Sigma _0^*$ is strictly positive, note first that $\Sigma ^*_{11} = \widehat{\Sigma } >0$ by assumption. To conclude, since $\Sigma >0$, it is enough to note that

$$\begin{aligned} \Sigma ^*_{22}-\Sigma ^*_{21} (\Sigma ^*_{11})^{-1}\Sigma ^*_{12} \, = \, \Sigma _{22}-\Sigma _{21}\Sigma _{11}^{-1}\Sigma _{12}. \end{aligned}$$

Finally, the relation ${\mathcal {I}}(\Sigma _0^*||\Sigma )={\mathcal {I}}(\widehat{\Sigma }||\Sigma _{11})$ is Equation (9.6) adapted to the present situation. The Pythagorean rule follows from this relation and Equation (9.7). $\square$

### *Proof of Proposition 3.7*

(*Second partial minimization*). We adhere to the setting and the notation of Proposition 9.4. Identify ${\mathbb {P}}={\mathbb {P}}_{XY}$ with the normal distribution $N(0, \Sigma )$ and ${\mathbb {Q}}={\mathbb {Q}}_{XY}$ with the normal $N(0, \Sigma _1)$, where $\Sigma _1\in {\varvec{\Sigma }}_1$. The optimal ${\mathbb {Q}}^*={\mathbb {Q}}^*_{XY}$ is again normal and specified by its (conditional) mean and covariance matrix. Since ${\mathbb {Q}}^*_{Y_i|X}={\mathbb {P}}_{Y_i|X}$ for all *i*, we have $\mathbb {E}_{{\mathbb {Q}}^*}[Y|X]=\mathbb {E}_{{\mathbb {P}}}[Y|X]=\Sigma _{12}\Sigma _{22}^{-1}X$; moreover, ${\mathbb {Q}}^*_X = {\mathbb {P}}_X$. Hence we find

$$\begin{aligned} \Sigma ^*_{12} = \mathbb {E}_{{\mathbb {Q}}^*} YX^\top = \mathbb {E}_{{\mathbb {Q}}^*}\mathbb {E}_{{\mathbb {Q}}^*}[Y|X]X^\top =\mathbb {E}_{{\mathbb {P}}}\mathbb {E}_{{\mathbb {P}}}[Y|X]X^\top =\Sigma _{12}. \end{aligned}$$

Furthermore, under ${\mathbb {Q}}^*$, the $Y_i$ are conditionally independent given *X*. Then ${\mathbb {C}}\hbox {ov}_{{\mathbb {Q}}^*}(Y_i,Y_j|X)=0$, for $i\ne j$, whereas $\mathbb {V}\mathrm{ar}_{{\mathbb {Q}}^*}(Y_i|X)=\mathbb {V}\mathrm{ar}_{{\mathbb {P}}}(Y_i|X)$, which is the *ii*-element of $\widetilde{\Sigma }_{11}:=\Sigma _{11}-\Sigma _{12}\Sigma _{22}^{-1}\Sigma _{21}$, from which it follows that ${\mathbb {C}}\hbox {ov}_{{\mathbb {Q}}^*}(Y|X)=\Delta (\widetilde{\Sigma }_{11}).$ We can now evaluate

$$\begin{aligned} \Sigma ^*_{11} = {\mathbb {C}}\hbox {ov}_{{\mathbb {Q}}^*}(Y)&= \mathbb {E}_{{\mathbb {Q}}^*}YY^\top \\&= \mathbb {E}_{{\mathbb {Q}}^*}\Big ( {\mathbb {E}\,}_{{\mathbb {Q}}^*}[Y|X]\,{\mathbb {E}\,}[Y|X]^\top + {\mathbb {C}}\hbox {ov}_{{\mathbb {Q}}^*}(Y|X) \Big ) \\&=\mathbb {E}_{{\mathbb {Q}}^*}\Big (\Sigma _{12}\Sigma _{22}^{-1}XX^\top \Sigma _{22}^{-1}\Sigma _{21} + \Delta (\widetilde{\Sigma }_{11}) \Big ) \\&=\Sigma _{12}\Sigma _{22}^{-1}\Sigma _{21} + \Delta (\widetilde{\Sigma }_{11}). \end{aligned}$$

The Pythagorean rule follows from the general result of Proposition 9.4. $\square$

### *Proof of Proposition 3.10*

(*Constrained second partial minimization*). We can still apply Lemma 9.3 and Proposition 9.4, with the proviso that the marginal distribution of *X* is fixed at some ${\mathbb {Q}}^0_X$. The optimal distribution ${\mathbb {Q}}^*_{XY}$ will therefore take the form ${\mathbb {Q}}^*_{XY}= \prod _i {\mathbb {P}}_{Y_i|X} {\mathbb {Q}}^0_X$.

Turning to the explicit computation of the optimal normal law, inspection of the proof of Proposition 3.7 reveals that under ${\mathbb {Q}}^*$ we have $\mathbb {E}_{{\mathbb {Q}}^*} YX^\top = \Sigma _{12}\Sigma _{22}^{-1}Q_0^\top Q_0$ and

$$\begin{aligned} {\mathbb {C}}\hbox {ov}_{{\mathbb {Q}}^*}(Y)=\Delta (\widetilde{\Sigma }_{11})+ \Sigma _{12}\Sigma _{22}^{-1}Q_0^\top Q_0\Sigma _{22}^{-1}\Sigma _{21}, \end{aligned}$$

thus completing the proof. $\square$

### *Proof of Proposition 4.3*

*(Update rule for*${\mathcal {H}}_t$). From Equation (4.5) one immediately gets

10.1 $$\begin{aligned} {\mathcal {H}}_{t+1}= H_{t+1}H_{t+1}^\top = \widehat{\Sigma }({\mathcal {H}}_t+D_t)^{-1}H_tR_t^{-1}H_t^\top ({\mathcal {H}}_t+D_t)^{-1}\widehat{\Sigma }. \end{aligned}$$

The key step in the proof is an application of the elementary identity, see e.g., Exercise 16(h) of Chapter 5 in Searle (1982),

$$\begin{aligned} (I+H^\top P H)^{-1}H^\top =H^\top (I+PHH^\top )^{-1}, \end{aligned}$$

valid for all *H* and *P* of appropriate dimensions for which both inverses exist. We have already seen that $R_t$ is invertible and of the type $I+HPH^\top$. Following this recipe, we compute

$$\begin{aligned} R_t^{-1}H_t^\top&= H_t^\top \big (I-({\mathcal {H}}_t+D_t)^{-1}{\mathcal {H}}_t +({\mathcal {H}}_t+D_t)^{-1}\widehat{\Sigma }({\mathcal {H}}_t+D_t)^{-1}{\mathcal {H}}_t\big )^{-1}\\&= H_t^\top \big ( ({\mathcal {H}}_t+D_t)^{-1}D_t+({\mathcal {H}}_t+D_t)^{-1}\widehat{\Sigma }({\mathcal {H}}_t+D_t)^{-1}{\mathcal {H}}_t\big )^{-1}\\&= H_t^\top \big (D_t + \widehat{\Sigma }({\mathcal {H}}_t+D_t)^{-1}{\mathcal {H}}_t\big )^{-1} ({\mathcal {H}}_t+D_t). \end{aligned}$$

Insertion of this result into (10.1) yields (4.7). $\square$

### *Proof of Proposition 6.6*

*(Update rule for*${\mathcal {H}}_t$, *singular case).* It is sufficient to show this for one iteration. We start from Equation (4.7) with $t=0$ and compute the value of ${\mathcal {H}}_1$. To that end we first obtain under the present assumption an expression for the matrix $({\mathcal {H}}_0+D_0)^{-1}{\mathcal {H}}_0$. Let $P=I-H_{20}^\top (H_{20}H_{20}^\top )^{-1}H_{20}$. It holds that

10.2 $$\begin{aligned} ({\mathcal {H}}_0+D_0)^{-1}{\mathcal {H}}_0= \begin{pmatrix} (\widetilde{D}_0 +H_{10}PH_{10}^\top )^{-1}H_{10}PH_{10}^\top &{} 0 \\ (H_{20}H_{20}^\top )^{-1}H_{20}H_{10}^\top (\widetilde{D}_0 +H_{10}PH_{10}^\top )^{-1}\widetilde{D}_0 &{} I \end{pmatrix}, \end{aligned}$$

as one can easily verify by multiplying this equation by ${\mathcal {H}}_0+D_0$. We also need the inverse of $D_0+\widehat{\Sigma }({\mathcal {H}}_0+D_0)^{-1}{\mathcal {H}}_0$, postmultiplied with $\widehat{\Sigma }$. Introduce $U=\widetilde{D}_0+\widetilde{\Sigma }_{11}(H_{10}PH_{10}^\top +\widetilde{D}_0)^{-1}H_{10}PH_{10}^\top$ and

$$\begin{aligned} V=\widehat{\Sigma }_{22}^{-1}\widehat{\Sigma }_{21}(H_{10}PH_{10}^\top + \widetilde{D}_0)^{-1}+ (H_{20}H_{20}^\top )^{-1}H_{20}H_{10}^\top (H_{20}H_{20}^\top )^{-1}\widetilde{D}_0. \end{aligned}$$

It results that

10.3 $$\begin{aligned} \big (D_0+\widehat{\Sigma }({\mathcal {H}}_0+D_0)^{-1}{\mathcal {H}}_0\big )^{-1}\widehat{\Sigma }= \begin{pmatrix} U^{-1}\widetilde{\Sigma }_{11} &{} 0 \\ -VU^{-1}\widetilde{\Sigma }_{11}+\widehat{\Sigma }_{22}^{-1}\widehat{\Sigma }_{21} &{} I \end{pmatrix}. \end{aligned}$$

Insertion of the expressions (10.2) and (10.3) into (4.7) yields the result. The equations for the stationary points follow as before. $\square$

## Footnotes

1 Note that, since $\widehat{\Sigma }>0$, the I-divergence ${\mathcal {I}}(\widehat{\Sigma } || HH^\top +D)$ is finite if and only if $HH^\top +D$ is invertible. This condition will always be assumed, without real loss of generality since the problem is to minimize the I-divergence.

2 For any non-negative definite matrix $M\ge 0$ a square root is any matrix *N* such that $M=NN^\top$. In general the square root is not unique, but if $M>0$ the symmetric square root is unique. A common notation for a square root of *M* is $M^{1/2}$.
